# Comparison of Meconium DNA Extraction Methods for Use in Microbiome Studies

**DOI:** 10.3389/fmicb.2018.00270

**Published:** 2018-02-20

**Authors:** Lisa F. Stinson, Jeffrey A. Keelan, Matthew S. Payne

**Affiliations:** Division of Obstetrics and Gynaecology, The University of Western Australia, Perth, WA, Australia

**Keywords:** DNA extraction, meconium, microbiome, PCR inhibitors, contamination

## Abstract

The establishment of human gut microbiota commences initially *in utero*. Meconium—the first fecal material passed after birth—can be used to study fetal gut contents; however, processing meconium samples for microbiome studies presents significant technical challenges. Meconium hosts a low biomass microbiome, is tar-like in texture and contains high concentrations of PCR inhibitors. This study aimed to evaluate four different DNA extraction methods to elucidate the most effective method for bacterial DNA recovery and sequencing analysis from first-pass meconium. Samples from five infants were collected and processed using the following extraction kits: (1) Qiagen QIAamp DNA Stool Mini (QS); (2) Qiagen QIAamp DNA Microbiome (QM); (3) MoBio PowerSoil (PS); (4) MoBio MagAttract PowerMicrobiome (PM). Additionally, Kit PM was employed with a double inhibitor removal treatment (IRT) step (PM2). Bacterial DNA recovery was assessed by qPCR. Any PCR inhibition in samples was measured by spiking DNA eluates with 0.1 ng of pure *Streptococcus agalactiae* (GBS) DNA followed by qPCR quantitation. Kit PM yielded the highest average total DNA yield (79.3 ng per gram of meconium). Samples extracted with kit PS had the highest detectable levels of 16S rRNA gene by qPCR. The ability of each kit to overcome PCR inhibition varied, with qPCR on GBS-spiked DNA from kits QS, QM, PS, and PM recovering 87.1, 91.0, 88.8, and 37.9% GBS DNA, respectively. Double IRT improved the performance of kit PM, increasing GBS recovery to 56.5%. However, once DNA yield was normalized to the level recovered with the other kits 100% of GBS DNA was detected, suggesting that levels of PCR inhibitors are related to DNA yield from kit PM. Ion Torrent 16S rRNA gene sequencing revealed a high level of inter-kit variation in meconium microbiome structure. In particular, kit QM showed a bias toward extracting Firmicute DNA, while the other kits extracted primarily Proteobacterial DNA. Choice of extraction kit greatly impacts on the ability to extract and detect bacterial DNA in meconium and on the microbiome community structure generated from these samples.

## Introduction

Traditionally, the establishment of human gastrointestinal (GI) microbiota has been seen as commencing at birth; however, increasing evidence suggests that the seeding process actually occurs initially *in utero* (Collado et al., 2016; Stinson et al., 2016). The GI microbiome plays a vital role in host health, with increasing evidence emerging that the disruption of this community may underpin a number of non-communicable diseases (Debarry et al., 2007; Moreno-Indias et al., 2014; Thorburn et al., 2015). For example, aberrations to the early-life GI microbiota may underpin the risk of asthma (Arrieta et al., 2015; Thorburn et al., 2015), allergies (Bunyavanich et al., 2016; Fujimura et al., 2016), and Crohn's disease (Gevers et al., 2014) later in life. To understand the formation of this microbial community, we must understand the origin and composition of the GI microbiome at birth, as this community may influence later colonization patterns via the founder effect. Additionally, the fetal GI microbiota may play a role in prenatal immune programming (Kaplan et al., 2011; Madan et al., 2012; Gosalbes et al., 2013; Hu et al., 2013; Romano-Keeler and Weitkamp, 2015).

First pass meconium can be analyzed as a non-invasive method of assessing fetal GI tract contents; however, there are a number of problems with processing this sample type. Meconium hosts a diverse, but low biomass microbiome (Jimenez et al., 2008; Gosalbes et al., 2013; Hu et al., 2013; Del Chierico et al., 2015; Collado et al., 2016). As a point of comparison, previously published work suggests that meconium yields 0.2 ± 0.4 ng of prokaryotic DNA per mg of meconium, compared with 16.6 ± 6.4 ng of prokaryotic DNA per mg of stool at 1 year of age (Wampach et al., 2017). The low yield of bacterial DNA from meconium is further complicated by its high concentrations of PCR inhibitors (Villanueva et al., 2000; Hansen et al., 2015). Meconium is a unique substance, and not stool in the traditional sense. It is not the excretion of waste products from digestion, but an accumulation of bile acids, pancreatic secretions, epithelial cells, and the residue of swallowed amniotic fluid. Meconium begins to form at the end of the first trimester of pregnancy, and is usually expelled by the infant within its first postnatal days (although in some cases the first meconium is passed before or during birth). Although the PCR inhibitors present meconium have never been isolated and identified, they are likely to include bile salts and acids (which are known to be strong inhibitors of PCR reactions; Al-Soud et al., 2005), glycolipids (Karlsson and Larson, 1978; which mimic the structure of nucleic acids), and urea originating from the amniotic fluid (which degrades polymerases; Schrader et al., 2012). A previous study has shown that PCR recovery of bacterial DNA from meconium can be as low as 10% (Hansen et al., 2015). Additionally, meconium is tar-like in texture and difficult to dissolve, adding further barriers to efficient DNA extraction. Thus, it is imperative to optimize and standardize DNA extraction methods for meconium samples.

While there is an overarching agreement in the literature that the meconium microbiome has a unique constitution, dominated by bacteria of the Proteobacteria and Firmicutes phyla, there is widespread lack of agreement in studies regarding the abundance and composition of meconium microbiota. In particular, the percent of colonized vs. sterile meconium varies greatly from study to study. Some authors have found 100% of meconium samples studied to be colonized, while others have found as little as 67% (Jimenez et al., 2008; Gosalbes et al., 2013; Hu et al., 2013; Ardissone et al., 2014; Hansen et al., 2015). Hansen et al., in their study of the meconium microbiome in a cohort of 15 neonates, found that they were only able to recover bacterial DNA from 1 patient using PCR. To confirm the sterility of the other samples, fluorescent *in situ* hybridization (FISH) was performed with probes specific for Bifidobacterium, Bacteroides-Prevotella, *Lactobacillaceae*/*Enterococcaceae, Enterobacteriaceae, Streptococcaceae, Staphylococcaceae*, and *Enterococcaceae*. Their FISH analysis revealed that 10 of the supposedly sterile samples were in fact colonized by 2–5 families of bacteria. This study in particular highlights the difficulties researchers face in analyzing the meconium microbiota by PCR to produce meaningful, unbiased and reproducible results.

Previous studies have compared commercially available DNA extraction kits for use in extracting bacterial DNA from stool for microbiome analysis (Nelson et al., 2010; Smith et al., 2011; Claassen et al., 2013; Mirsepasi et al., 2014). However, a comparable analysis for meconium is lacking. The International Human Microbiome Standards (IHMS) consortium provides two standardized protocols for extraction of microbial DNA from stool samples, including a modified protocol for the Qiagen QIAamp DNA Stool Kit (Dore et al., 2015). No standard operating procedures have been published for microbiome work on meconium. Given the unique qualities of this sample type, and the inherent problems with DNA yield and external contamination of low biomass samples, there is a need for the development of a meconium-specific standard protocol.

The Qiagen QIAamp DNA Stool Kit Mini (kit QS) and the MoBio Power Soil kit (kit PS) are widely used for DNA extraction from stool; more recently, both Qiagen and MoBio have released microbiome kits [QIAamp DNA Microbiome kit (kit QM) and MoBio MagAttract PowerMicrobiome DNA/RNA kit (kit PM)]. Kit QM can be used to selectively recover prokaryotic DNA for microbiome analysis, while kit PM can recover both DNA and RNA to allow analysis of RNA viruses.

Using first pass meconium samples, the present study aimed to compare these four DNA extraction methods to assess bacterial DNA recovery, removal of PCR inhibitors, and resulting bacterial community structures in order to define the optimal extraction method for use in meconium microbiome studies.

## Materials and methods

### Sample collection

First pass meconium was collected from five infants born by elective Cesarean section to healthy mothers at King Edward Memorial Hospital, Subiaco, Western Australia with the approval of the Human Research Ethics Committee of the Western Australian Department of Health's Women and Newborns Health Service (2015026EW). All samples were passed within 12 h of birth (mean = 6.4 h) and processed within an hour of being passed. Whole nappies were removed from the infants by gloved midwives, de-identified and placed in sterile transport bags. Samples were then taken from the nappies in a level two biosafety cabinet using aseptic techniques. For each meconium sample, five aliquots of 200 ± 3 mg were taken and stored at −20°C until extraction (<1 week). To limit the possibility of external contamination from the nappy or infant's skin, the outer layer of the meconium was removed using a sterile scalpel. An inner portion of meconium was then retrieved using a sterile syringe, then immediately distributed into PCR safe tubes for extraction.

### Tween-80 treatment

The tar-like consistency of meconium does not lend itself easily to DNA extraction. Meconium can block filters in spin column extractions and cause bead carryover into the eluate in magnetic bead-based extractions. After several failed extraction attempts, we identified a published method of meconium solubilization using a 10% Tween-80 solution (Coran et al., 2012). Samples were mixed with 1 ml sterile 10% Tween 80 to achieve liquefaction, then vortexed horizontally for 20 min (MoBio Vortex-Genie 2, speed setting 7) and centrifuged at 40,000 × g for 5 min. The supernatant was discarded and the pellet resuspended in 1 ml UltraPure water. The samples were again centrifuged at 40,000 × g for 5 min. The supernatant was again discarded and the pellet was immediately processed with the appropriate extraction kit.

### Extractions

One 200 mg aliquot of meconium from each infant was processed with each extraction method. The extraction kits used are described in Table 1. Extractions with kit PM were processed on the King Fisher Duo platform. All extractions were performed according to the manufacturer's instructions, with the exception of kit QS, for which a lysing temperature of 90°C was used instead of 70°C, as recommended by the manufacturer to process difficult to lyse samples. One aliquot of each meconium sample was processed with kit PM following the manufacturer's instructions, and a second set of aliquots was processed with a double inhibitor removal (IRT) step for all but one sample (PM2), for which there was insufficient remaining sample. All samples were eluted in 100 μl of UltraPure water. An extraction control consisting of 250 μl of sterile DNA-free water was used for each kit.

**Table 1:** Summary of DNA extraction kit characteristics.

**Extraction kit**	**Manufacturer**	**Abbreviation**	**Principle**	**Bead beating component?**	Cost per sample (USD)^*^	Completion time (hours)^*^
Stool Mini	Qiagen	QS	Spin column	No	$4.58	2.25
Microbiome	Qiagen	QM	Spin column	Yes	$10.67	4.75
Power Soil	MoBio	PS	Spin column	Yes	$5.48	1.75
Power Microbiome	MoBio	PM	Magnetic beads	Yes	$5.18	2.50
Power Microbiome double IRT	MoBio	PM2	Magnetic beads	Yes	$5.18	2.75

* *Cost and completion time based on processing 5 samples and 1 extraction control per batch. Completion time includes time taken to pre-treat samples with Tween-80*.

### DNA yield

DNA yield was assessed using the Qubit® dsDNA HS Assay kit with a Qubit® 2.0 fluorometer. The limit of detection was 10 pg/μl.

### Quantification of human DNA in meconium samples

Levels of human DNA present in each meconium sample were assessed via qPCR for the human β globin gene, as previously described (Klaassen et al., 2003). A standard curve was constructed using EpiTech control human DNA (Qiagen) and PCR was carried out in 20 μl reactions containing 5 μl of template or water (negative template control), 1X TaqMan Fast Advanced Master Mix (Applied Biosystems), 0.1 μM each of the forward (5′-GGGCAACGTGCTGGTCTG-3′) and reverse (5′-AGGCAGCCTGCACTGGT-3′) primers, 0.25 μM of probe (5′-FAM-CTGGCCCATCACTTTGGCAAAGAA-TAMRA-3′), and 4.2 μl of water. The PCR amplification program consisted of an initial heating step of 95°C for 20 s, followed by 40 cycles of 95°C for 1 s and 60°C for 20 s. PCR reactions were performed on a ViiA7 Real-Time PCR System (Life Technologies). All samples and controls were run in duplicate.

### 16S rRNA qPCR

Real-time PCR was performed to compare relative levels of bacterial DNA recovery between extraction methods. The V6 region of the 16S rRNA gene was amplified as previously described (Yang et al., 2002) in 20 μl reactions containing 5 μl of template or water (negative template control), 1X TaqMan Fast Advanced Master Mix (Applied Biosystems), 0.1 μM each of the forward (5′-TGGAGCATGTGGTTTAATTCGA-3′) and reverse (5′- TGCGGGACTTAACCCAACA-3′) primers, 0.25 μM of probe (5′-FAM-CACGAGCTGACGACARCCATGCA-BHQ1-3'), and 4.2 μl of water. All samples and controls were run in duplicate.

### Inhibitor assessment

In order to quantify the effect of PCR inhibitors present in meconium, undiluted, purified DNA from each kit was spiked with 0.1 ng of purified *Streptococcus agalactiae* (Group B Streptococcus—GBS) DNA. By comparing the amount of GBS DNA recovered from spiked meconium samples to the amount of GBS DNA recovered from spiked extraction controls we were able to assess the presence of PCR inhibitors remaining after extraction with each kit. Routine culture-based testing for GBS in the vaginal tracts of each participating mother were negative. In addition, all meconium samples were confirmed as negative for GBS DNA by molecular screening (GBS targeted qPCR, as described below) prior to the spiking experiments.

Five microliter of extracted meconium DNA was spiked with 0.1 ng of pure GBS DNA. This eluate + GBS DNA mix was then used as the template for qPCR. GBS DNA levels were quantified using the dltS primer/probe set as previously described (Furfaro et al., 2017). PCR was carried out in 20 μl reactions containing 5 μl of template or water (negative template control), 1X TaqMan Fast Advanced Master Mix (Applied Biosystems), 0.1 μM each of the forward and reverse primers, 0.25 μM of probe, and 4.2 μl of water. PCR conditions were as described above. A standard curve was used to quantify the percent recovery of GBS DNA from each sample. All samples and controls were run in duplicate.

### Endpoint PCR

Endpoint PCR was performed to amplify the V3-V4 region of the 16S rRNA gene for sequencing. The primers used were 341F (5'-CCTACGGGNGGCWGCAG-3') and 785R (5′-GACTACHVGGGTATCTAATCC-3′), previously validated as providing optimal coverage of the domain Bacteria for a 400–1,000 bp amplicon (Klindworth et al., 2013). PCR was carried out in 50 μl reactions containing 5 μl of template or water (negative template control), 1X 360 PCR buffer (ABI), 2 mM MgCl_2_, 200 μM dNTPs, 1.25U of Taq, 0.5 μM each of the forward and reverse primers, and 29.25 μl of water. The PCR amplification program consisted of an initial heating step at 94°C for 3 min; 40 cycles of 95°C for 30 s, 55°C for 30 s, and 72°C for 1 min; and a final extension step of 72°C for 7 min. PCR reactions were performed on an Applied Biosystems Veriti Thermal Cycler. PCR products were visualized on a QIAxcel automated electrophoresis system using a DNA high resolution gel cartridge (run parameters 0M500) to confirm the presence and size of amplicons.

### Ion torrent sequencing

For NGS library preparation, the PCR products were purified using the Agencourt AMPure XP Reagent (Beckman Coulter) following the manufacturer's protocol and re-suspended in 20 μl of Low TE buffer (0.1 mM EDTA, 10 mM Tris-HCl pH 8). The purified amplicons were quantified using the Qubit Fluorometer 2.0 and Qubit dsDNA Broad Range Assay Kit (Thermo Fisher Scientific) according to the manufacturer's protocol. To enable sequencing adaptor and sample indexing barcode ligation, the purified PCR products (100 ng) were first blunt-ended using the End Repair Enzyme Mix (Thermo Fisher Scientific) according to the manufacturer's protocol. The Ion P1 Adaptor and Ion Xpress Barcodes 1–26 were ligated to the amplicons using the DNA Ligase Mix (Thermo Fisher Scientific) according to the manufacturer's protocol. The adaptor-ligated libraries were purified using the Agencourt AMPure XP Reagent, re-suspended in 20 μl of Low TE Buffer and amplified using the Platinum PCR SuperMix High Fidelity and Library Amplification Primer Mix. The thermal cycling conditions consisted of an initial denaturation at 95°C for 5 min, followed by 5 cycles of 95°C for 15 s, 58°C for 15 s and 70°C for 1 min. The libraries were again purified using the Agencourt AMPure XP Reagent and quantified using the Qubit dsDNA HS Assay Kit. Each library was adjusted to 100 pM in Low TE Buffer and combined in an equimolar ratio to ensure equal representation of each barcoded library in the sequencing reaction.

Automated template preparation using isothermal amplification technology and chip loading was performed using the Ion 520 and 530 ExT Kit on the Ion Chef System (Thermo Fisher Scientific). A 50 μl aliquot of the 100 pM pooled library was added to the Ion S5 ExT Reagents cartridge for templating onto Ion Sphere Particles (ISPs) and loading into an Ion 520 Chip. The loaded Ion 520 Chip was sequenced for 1,300 flows using the Ion S5 ExT Sequencing Kit on an Ion S5 Sequencer with Torrent Suite Software version 5.2.2 using Default Calibration (Thermo Fisher Scientific).

### Sequencing data processing

Sequences generated from Ion Torrent sequencing of 16S rRNA gene PCR amplicons were analyzed using default setting on the open-source software Quantitative Insights into Microbial Ecology (QIIME) version 1.9.1 (Caporaso et al., 2010b). Chimeras, low quality reads (Q<25), and reads of < 400 bp in length were removed. Reads were assigned to operational taxonomic units using the *pick_open_reference_otus.py* command with default parameters using the UCLUST method (Edgar, 2010). OTUs occurring only once (singletons) or that failed to align using PyNAST were removed (Caporaso et al., 2010a). Relative diversity analyses were generated using the command *core_diversity_analyses.py*. Raw sequence reads have been submitted to the Sequence Read Archive (accession number SRP128962).

## Results and discussion

### DNA yield

**Figure 1:**
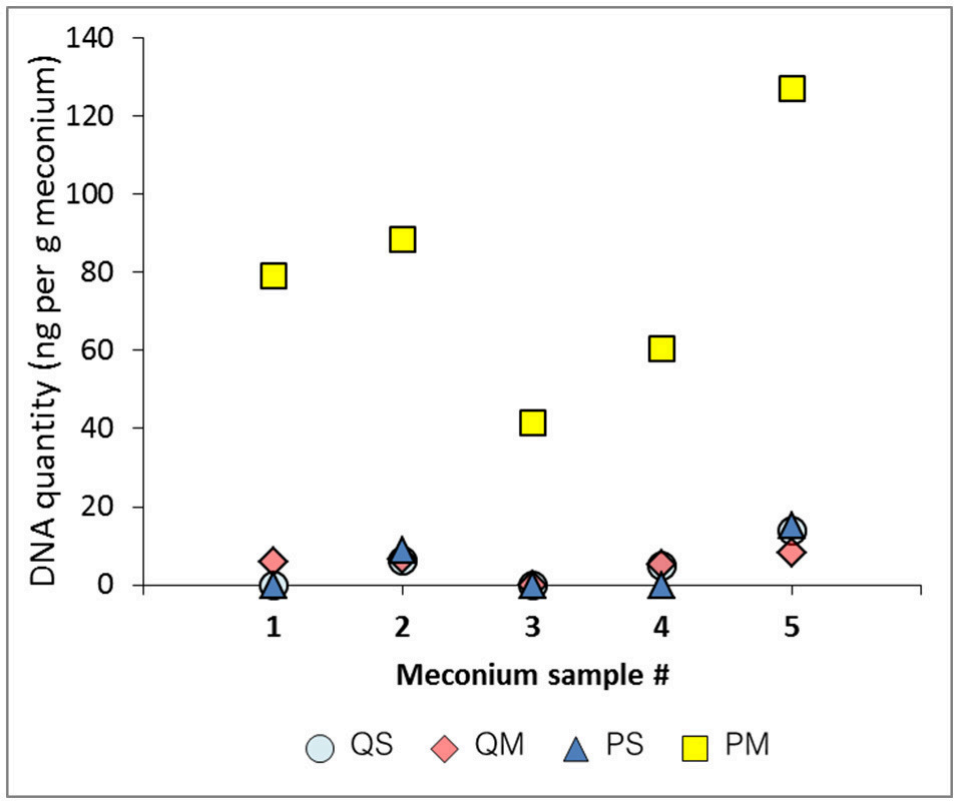
DNA quantitation (ng/g meconium) for each meconium sample using various extraction methods.

DNA yield varied between meconium samples, but was even more variable according to extraction method (Figure 1). Kit PM produced markedly (~10-fold) higher mean DNA yield (80 ng/g meconium) compared to the other kits (QS = 5 ng/g meconium, QM = 5.15 ng/g meconium, PS = 4.85 ng/g meconium), suggesting a superior extraction efficiency. This result is particularly interesting as kit QM isolates only prokaryotic DNA, while kit PM isolates all DNA (i.e., bacterial and human). This may suggest that kit PM recovers a high quantity of human DNA from meconium samples. However, it may also indicate that kit PM is simply able to recover more DNA than kit QM in general. All negative extraction controls yielded DNA below the limit of detection (10 pg/μl, data not shown). Our extractions yielded significantly less total DNA than those reported in meconium by Wampach et al. (mean: 200 ng/g; Wampach et al., 2017) who employed an unspecified pre-processing step, followed by a modified kit PS protocol.

### Quantification of human DNA in meconium samples

To assess the extent to which contaminating host DNA influenced the results from the quantification of total DNA, we performed a qPCR for human β-globin DNA. Levels of human DNA were very low, below the limit of detection (0.5 pg/μl) in several samples (1/5 from kit QS, 3/5 for kit QM, 5/5 for kit PS, and 2/5 for kit PM). Human DNA made up 15.8% of total extracted DNA from kit QS, 4.8% of total extracted DNA from kit QM, and 1.3% of total extracted DNA from kit PM. All extraction controls were below the limit of detection. Human DNA thus makes up a minor proportion of the total DNA content of meconium, which suggests that the high levels of total DNA seen in eluates from kit PM reflect greater microbial DNA extraction efficiency than the other kits, not human DNA contamination.

### 16S rRNA qPCR

Contaminating bacterial DNA is ubiquitous in DNA extraction kits and other laboratory reagents (Salter et al., 2014). Such contamination can be a major confounding factor in metagenomic studies of low-biomass samples such as meconium. For this reason, a negative extraction control was processed alongside our meconium samples to provide a point of reference for bacterial DNA contamination.

**Figure 2:**
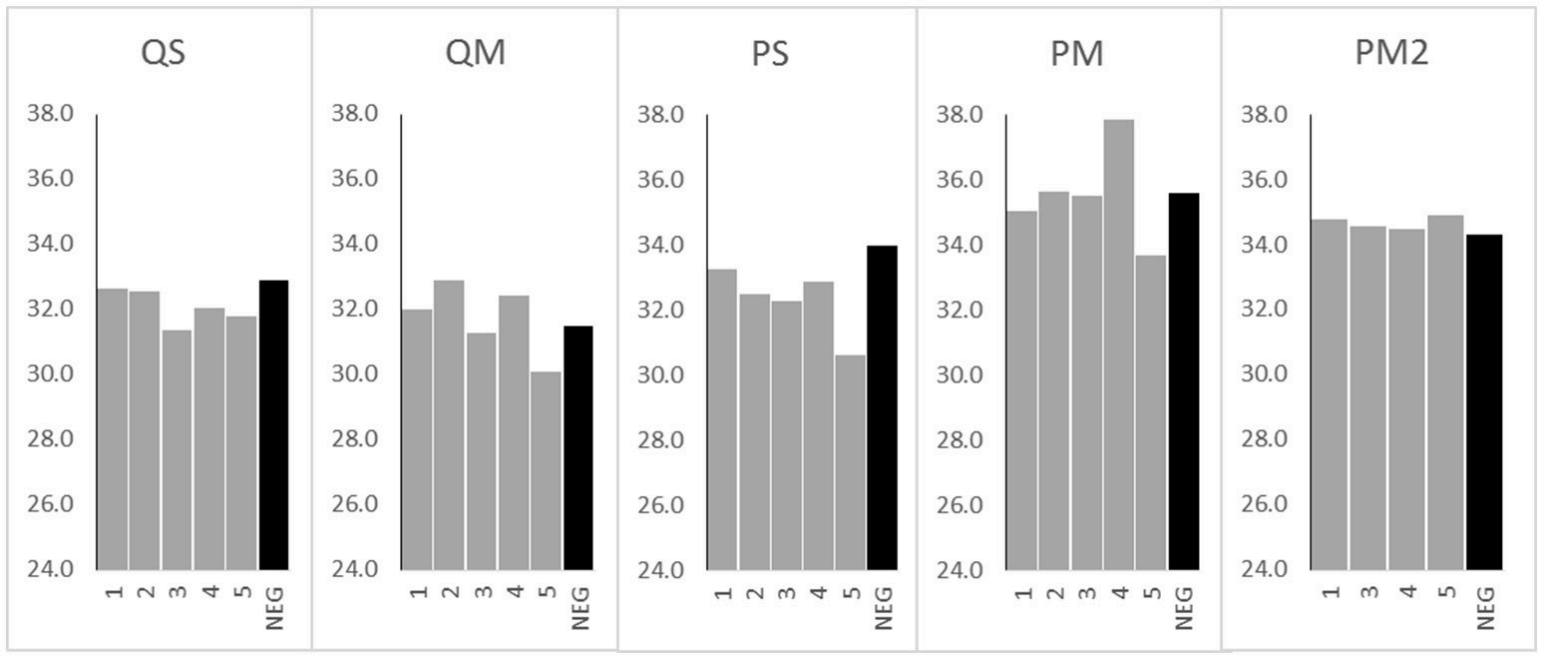
16S rRNA gene Ct values from meconium samples (*n* = 5 for kits QS, QM, PS, and PM, *n* = 4 for kit PM2) and negative extraction controls (NEG) (*n* = 1 per kit) processed with various extraction kits. Sample numbers (1–5 or NEG) are displayed on the x axis. Ct values are displayed on the y axis.

The extraction controls had DNA levels below the limit of detection of the Qubit high sensitivity DNA quantitation assay (10 pg/μl). Mean cycle threshold (Ct) values for each negative extraction control were as follows: kit QS, 32.9; kit QM, 31.5; kit PS, 33.9; kit PM, 35.6; kit PM2, 34.3 (Figure 2, black bars). Kit PM returned the highest Ct value for its negative extraction control, suggesting the lowest amount of DNA contamination in the kit components. Two negative PCR controls were run alongside all samples and gave a mean Ct result of 33.6 ± 0.3 (data not shown). With kits QS and PS, sample extracts contained more DNA (lower Ct values) than negative extraction controls; this was not the case for samples processed with kits QM and PM. In the case of kits QS and QM, extraction control Ct values were lower than those of the negative PCR control (33.6), suggesting that these kits contributed a small amount of bacterial DNA contamination.

The greatest difference between mean Ct values of the negative extraction controls and samples was seen in kit PS, with a 1.7 cycle difference. Only 2/5 samples extracted with kit QM and only 3/5 samples extracted with kit PM yielded Ct values below the negative extraction control (Figure 2). Therefore, to assess the impact of PCR inhibitors in the amplification and detection of DNA with kit PM, a second extraction was performed using a double inhibitor removal treatment (IRT) step. This extraction method (PM2) did not appreciably lower the Ct value for the same sample set, suggesting that PCR inhibition remained a significant issue.

### Inhibitor assessment

**Figure 3:**
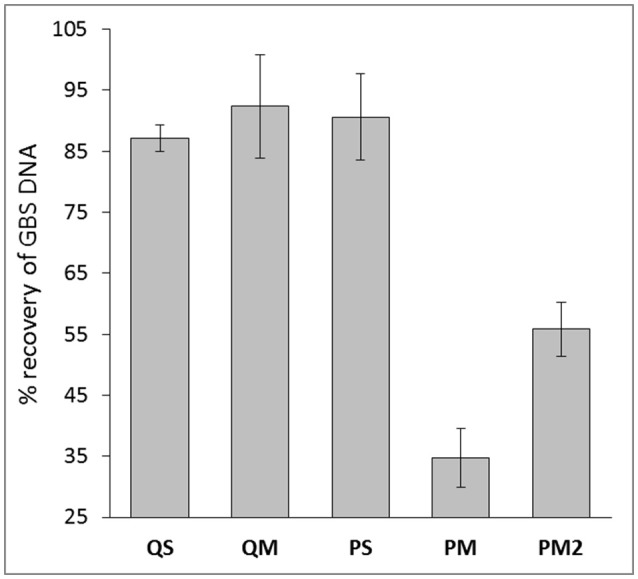
Percent recovery of GBS DNA with meconium extractions generated from various extraction kits. Data are mean ± SD.

Despite the use of PCR inhibitor removal steps, PCR inhibitors remained after processing with each of the four tested extraction kits. The least evidence of PCR inhibition was found in kit QM (median 9.0% inhibition), followed by kit PS (median 11.2% inhibition), and kit QS (median 12.9% inhibition; Figure 3). Kit PM performed poorly in this regard, with a median 62.1% inhibition. Addition of the double inhibitor removal step had modest impact, reducing the level of inhibition to a median 43.5% (PM2). However, this did not translate to improved qPCR results (Figure 2).

To further investigate the high levels of PCR inhibition associated with kit PM, we normalized DNA levels in each sample extracted with kit PM2 to the average levels of DNA in eluates produced with the other 3 kits (5 ng/g meconium). After normalization, we observed no PCR inhibition from PM2 eluates, suggesting that levels of inhibition are relative to levels of extracted DNA. Alternatively the inhibitors may have been diluted to an insignificant level.

Given the qPCR results and those of our DNA quantification, we concluded that kit PM achieves the highest yield of DNA but suffers from the greatest degree of PCR inhibition. However, this inhibition appears to be directly related to the high DNA yields and was completely resolved following dilution of DNA in line with levels extracted from the other three kits. It is possible that some PCR inhibitors in meconium are similarly charged to DNA, and since kit PM is magnetic-bead based, this would result in concurrent transfer of DNA and any remaining inhibitors into the eluates. We hypothesize that there is likely to be an optimal DNA dilution ratio that still maintains minimal PCR inhibition with kit PM, however, this may also be sample-specific, meaning that construction of a DNA dilution series and subsequent inhibitor assay screening could be a necessary step in DNA extraction protocols for meconium prior to downstream analyses with this kit. This will be explored in additional studies. Kit PM also has the advantage in that it is able to extract both DNA and RNA, allowing users to analyze the bacterial and viral component of the meconium microbiome. Kit PS also performed well, with low levels of inhibition from undiluted DNA as evident in 16S rRNA qPCR and GBS spiking experiments, however, the overall DNA yield from this kit were 16-fold less than that of kit PM.

### 16S rRNA sequencing

**Figure 4:**
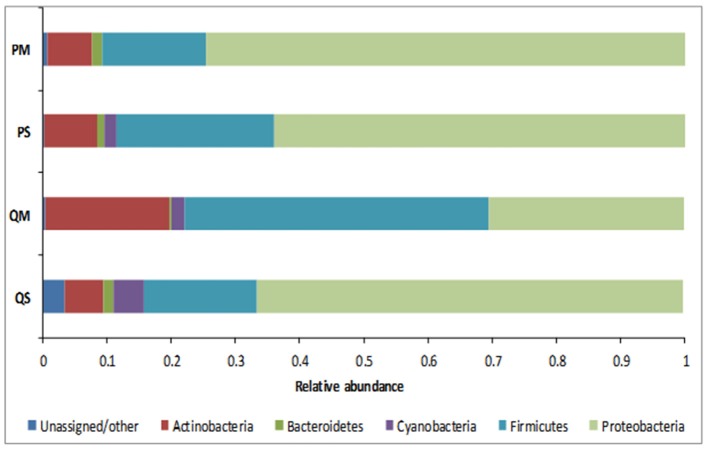
Relative abundance of OTUs in meconium samples (*n* = 5) after extraction with various kits at phylum level.

**Figure 5:**
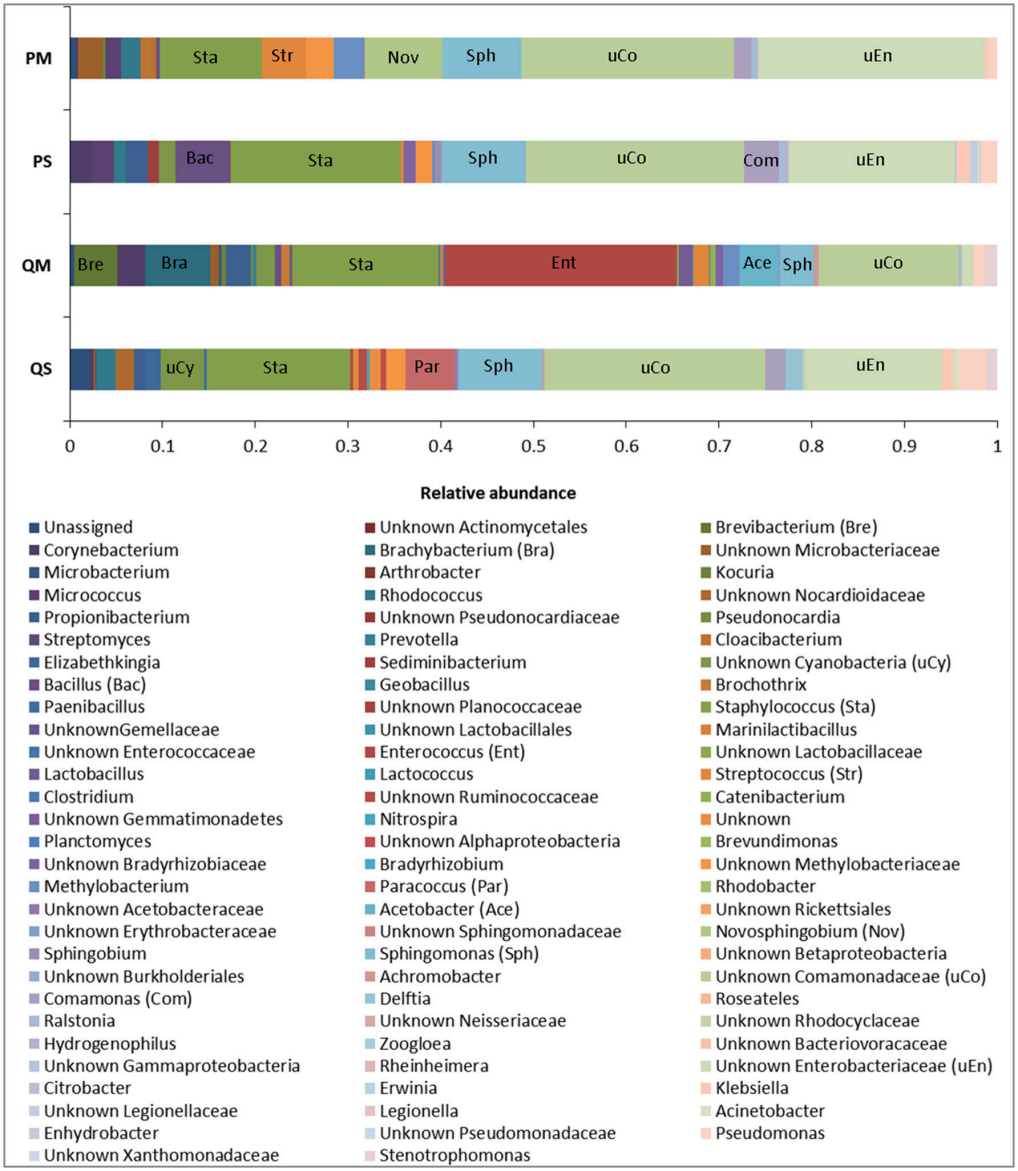
Relative abundance of OTUs in meconium samples (*n* = 5) after extraction with various kits at genus level. Major genera are labeled for identification purposes.

16S rRNA analysis showed that the meconium microbiome was dominated by sequences affiliated with bacteria of the Proteobacteria and Firmicutes phyla, as previously reported in numerous studies (reviewed in Stinson et al., 2016). However, in the present study a high level of inter-kit variation in meconium microbiome structure was observed (Figures 4, 5). In particular, amplicons generated from DNA extracted with kit QM consisted largely of sequences affiliated with Firmicutes (with a total of 47.4% of reads belonging to this phylum), while the other kits were mainly affiliated with Proteobacteria (Figure 4).

The microbial profiles generated after extraction with kits QS, PS, and PM were similar, with QS-extracted meconium DNA dominated by sequences affiliated with unknown *Comamonadaceae* (23.8%), *Staphylococcus* spp. (15.5%), unknown *Enterobacteriaceae* (14.7%), and *Sphingomonas* spp. (9.1%); PS-extracted meconium DNA was dominated by unknown *Comamonadaceae* (23.5%), *Staphylococcus* spp. (18.3%), unknown *Enterobacteriaceae* (17.9%), and *Sphingomonas* spp. (9.1%), while PM-extracted meconium DNA was dominated by unknown *Enterobacteriaceae* (24.4%), unknown *Comamonadaceae* (22.9%), *Staphylococcus* spp. (11.1%), and *Sphingomonas* spp. (8.5%). For kit QM, however, the microbial profile was dominated by sequences affiliated with *Enterococcus* spp. (25.1%), *Staphylococcus* spp. (15.8%), unknown *Comamonadaceae* (15.1%), and *Brachybacterium* spp. (7.1%) (Figure 5).

The major differences in microbial profiles between kit QM and the other kits may be due to the inclusion of a eukaryotic DNA removal step in kit QM. Prior to bacterial cell lysis, host cells are selectively lysed and DNA is enzymatically degraded. It is possible that some bacterial cells may be lysed during this step, for instance those attached to human cells, thus changing the bacterial community structure. However, amplicons generated from kit QM DNA produced more than twice as many sequences as the other kits (mean number of reads per sample: QM = 6282; PS = 3622; QS = 977; PM = 814). Kit QM also recovered the highest number of unique sequences at the genus level (14, compared to 8 from PS, 2 from PM, and 1 from QS). Thus, it seems unlikely that a large quantity or diversity of bacteria is lost in this step. Although the precise host DNA removal methods used in the kit are proprietary and not disclosed, previous studies have demonstrated that removal of human DNA through selective lysis of eukaryotic cells is not 100% efficient, and results in some loss of bacterial DNA (Hunter et al., 2011).

It is difficult to compare our results to the “true” meconium microbiome, as it is a poorly studied substance. Instead we tested the reproducibility of each extraction kit per-patient by quantifying its ability to recover the aggregate microbiome for each patient. All OTUs recovered from a single patient across all kits were pooled, and each kit was scored by its ability to recover this pooled microbiome for each patient. Kit QM recovered the highest percentage of OTUs per patient, 44–79%. Kit QS was able to recover 20–47%, kit PS recovered 33–55%, and kit PM recovered 12–47%. Using this rationale, it appears that kit QM is best able to extract the “true” meconium microbiome.

A number of OTUs were detected in our negative extraction controls (14 from kit QS, 38 from kit QM, 17 from kit PS, 23 from kit PM) and in our negative PCR controls (21) (Table 2). It is now well established that negative extraction and PCR controls contain trace amounts of microbial DNA (Salter et al., 2014; Weiss et al., 2014), and that contamination from extraction kits and laboratory reagents is a major confounding issue when working with low biomass samples such as meconium (Lauder et al., 2016). Fifty one OTU sequences were found only in meconium samples, not in negative extraction controls or PCR controls (Table 3). Thus, we can say with some certainty that the source of these bacterial sequences was meconium.

**Table 2:** Summary of OTUs detected in negative extraction controls (*n* = 1 per kit) and negative PCR controls (*n* = 2).

**OTU**	**Relative abundance**
**KIT QS**	
k__Bacteria;p__Proteobacteria;c__Betaproteobacteria;o__Burkholderiales;f__Comamonadaceae;g__	0.450292
k__Bacteria;p__Proteobacteria;c__Alphaproteobacteria;o__Sphingomonadales;f__Sphingomonadaceae;g__Sphingomonas	0.280298
k__Bacteria;p__Proteobacteria;c__Alphaproteobacteria;o__Rhizobiales;f__Methylobacteriaceae;g__	0.149829
k__Bacteria;p__Proteobacteria;c__Betaproteobacteria;o__Burkholderiales;f__Comamonadaceae;g__Comamonas	0.071184
k__Bacteria;p__TM7;Other;Other;Other;Other	0.033878
k__Bacteria;p__Proteobacteria;c__Gammaproteobacteria;o__Pseudomonadales;f__Moraxellaceae;g__Acinetobacter	0.005041
k__Bacteria;p__Proteobacteria;c__Betaproteobacteria;o__Burkholderiales;f__Oxalobacteraceae;g__Ralstonia	0.003831
k__Bacteria;p__Proteobacteria;c__Betaproteobacteria;o__Burkholderiales;f__Comamonadaceae;Other	0.001613
Unassigned;Other;Other;Other;Other;Other	0.00121
k__Bacteria;p__Firmicutes;c__Bacilli;o__Bacillales;f__Staphylococcaceae;g__Staphylococcus	0.00121
k__Bacteria;p__Proteobacteria;c__Alphaproteobacteria;o__Sphingomonadales;f__Sphingomonadaceae;g__	0.000605
k__Bacteria;p__Proteobacteria;c__Betaproteobacteria;o__Rhodocyclales;f__Rhodocyclaceae;g__	0.000605
k__Bacteria;p__Firmicutes;c__Bacilli;o__Lactobacillales;f__Streptococcaceae;g__Streptococcus	0.000202
k__Bacteria;p__Proteobacteria;c__Gammaproteobacteria;o__Enterobacteriales;f__Enterobacteriaceae;g__	0.000202
**KIT QM**	
k__Bacteria;p__Proteobacteria;c__Alphaproteobacteria;o__Rhodospirillales;f__Acetobacteraceae;g__Acetobacter	0.202392
k__Bacteria;p__Actinobacteria;c__Actinobacteria;o__Actinomycetales;f__Dermabacteraceae;g__Brachybacterium	0.137781
k__Bacteria;p__Actinobacteria;c__Actinobacteria;o__Actinomycetales;f__Brevibacteriaceae;g__Brevibacterium	0.091815
k__Bacteria;p__Actinobacteria;c__Actinobacteria;o__Actinomycetales;f__Corynebacteriaceae;g__Corynebacterium	0.079386
k__Bacteria;p__Firmicutes;c__Bacilli;o__Lactobacillales;f__Enterococcaceae;g__Enterococcus	0.064259
k__Bacteria;p__Firmicutes;c__Bacilli;o__Bacillales;f__Listeriaceae;g__Brochothrix	0.058865
k__Bacteria;p__Proteobacteria;c__Betaproteobacteria;o__Burkholderiales;f__Comamonadaceae;g__	0.057575
k__Bacteria;p__Proteobacteria;c__Alphaproteobacteria;o__Rhizobiales;f__Methylobacteriaceae;g__Methylobacterium	0.049367
k__Bacteria;p__Firmicutes;c__Bacilli;o__Lactobacillales;f__Lactobacillaceae;g__Lactobacillus	0.031191
k__Bacteria;p__Actinobacteria;c__Actinobacteria;o__Actinomycetales;f__Propionibacteriaceae;g__Propionibacterium	0.026853
k__Bacteria;p__Actinobacteria;c__Actinobacteria;o__Actinomycetales;f__Micrococcaceae;g__Rothia	0.022397
k__Bacteria;p__Firmicutes;c__Bacilli;o__Lactobacillales;f__Aerococcaceae;g__	0.021459
k__Bacteria;p__Firmicutes;c__Bacilli;o__Lactobacillales;f__Streptococcaceae;g__Streptococcus	0.015713
k__Bacteria;p__Proteobacteria;c__Alphaproteobacteria;o__Sphingomonadales;f__Sphingomonadaceae;g__Sphingomonas	0.01454
k__Bacteria;p__Proteobacteria;c__Gammaproteobacteria;o__Enterobacteriales;f__Enterobacteriaceae;g__	0.013368
k__Bacteria;p__Firmicutes;c__Bacilli;o__Lactobacillales;f__Aerococcaceae;g__Facklamia	0.011257
k__Bacteria;p__Actinobacteria;c__Actinobacteria;o__Actinomycetales;f__Kineosporiaceae;g__	0.01114
k__Bacteria;p__Firmicutes;c__Bacilli;o__Bacillales;f__Bacillaceae;g__Bacillus	0.010436
k__Bacteria;p__Proteobacteria;c__Alphaproteobacteria;o__Rhodospirillales;f__Acetobacteraceae;Other	0.010319
k__Bacteria;p__Proteobacteria;c__Gammaproteobacteria;o__Xanthomonadales;f__Xanthomonadaceae;g__Stenotrophomonas	0.009615
k__Bacteria;p__Proteobacteria;c__Deltaproteobacteria;o__Desulfovibrionales;f__Desulfohalobiaceae;g__	0.009381
k__Bacteria;p__Proteobacteria;c__Alphaproteobacteria;o__Rhizobiales;f__Methylobacteriaceae;g__	0.009264
k__Bacteria;p__Actinobacteria;c__Actinobacteria;o__Actinomycetales;f__Dietziaceae;g__Dietzia	0.009146
k__Bacteria;p__Proteobacteria;c__Gammaproteobacteria;o__Pseudomonadales;f__Moraxellaceae;g__	0.008208
k__Bacteria;p__Proteobacteria;c__Gammaproteobacteria;o__Pseudomonadales;f__Moraxellaceae;g__Enhydrobacter	0.007505
k__Bacteria;p__Firmicutes;c__Bacilli;o__Lactobacillales;f__;g__	0.007036
k__Bacteria;p__Actinobacteria;c__Actinobacteria;o__Actinomycetales;f__Intrasporangiaceae;g__	0.005629
k__Bacteria;p__Firmicutes;c__Bacilli;o__Lactobacillales;f__Enterococcaceae;g__	0.000938
k__Bacteria;p__Proteobacteria;c__Betaproteobacteria;o__Burkholderiales;f__Comamonadaceae;g__Comamonas	0.000821
Unassigned;Other;Other;Other;Other;Other	0.000821
k__Bacteria;p__Firmicutes;c__Bacilli;o__Bacillales;f__Staphylococcaceae;g__Staphylococcus	0.000352
k__Bacteria;p__Firmicutes;c__Bacilli;o__Lactobacillales;f__Enterococcaceae;Other	0.000235
k__Bacteria;p__Proteobacteria;c__Alphaproteobacteria;o__Sphingomonadales;f__Sphingomonadaceae;g__	0.000235
k__Bacteria;p__Proteobacteria;c__Betaproteobacteria;o__Burkholderiales;f__Comamonadaceae;Other	0.000235
k__Bacteria;p__Bacteroidetes;c__Flavobacteriia;o__Flavobacteriales;f__[Weeksellaceae];g__Elizabethkingia	0.000117
k__Bacteria;p__Cyanobacteria;c__4C0d-2;o__MLE1-12;f__;g__	0.000117
k__Bacteria;p__Proteobacteria;c__Betaproteobacteria;o__Rhodocyclales;f__Rhodocyclaceae;g__	0.000117
k__Bacteria;p__Proteobacteria;c__Gammaproteobacteria;o__Enterobacteriales;f__Enterobacteriaceae;g__Klebsiella	0.000117
**KIT PS**	
k__Bacteria;p__Proteobacteria;c__Betaproteobacteria;o__Burkholderiales;f__Comamonadaceae;g__	0.347242
k__Bacteria;p__Proteobacteria;c__Betaproteobacteria;o__Burkholderiales;f__Comamonadaceae;g__Delftia	0.151218
k__Bacteria;p__Cyanobacteria;c__4C0d-2;o__MLE1-12;f__;g__	0.127975
k__Bacteria;p__Bacteroidetes;c__Flavobacteriia;o__Flavobacteriales;f__Flavobacteriaceae;g__Capnocytophaga	0.105853
k__Bacteria;p__Proteobacteria;c__Alphaproteobacteria;o__Sphingomonadales;f__Sphingomonadaceae;g__Sphingomonas	0.07925
k__Bacteria;p__Proteobacteria;c__Betaproteobacteria;o__Burkholderiales;f__Comamonadaceae;g__Comamonas	0.055447
k__Bacteria;p__Bacteroidetes;c__Bacteroidia;o__Bacteroidales;f__Prevotellaceae;g__Prevotella	0.032484
k__Bacteria;p__Proteobacteria;c__Gammaproteobacteria;o__Pseudomonadales;f__Moraxellaceae;g__Acinetobacter	0.031364
k__Bacteria;p__Proteobacteria;c__Gammaproteobacteria;o__Pseudomonadales;f__Pseudomonadaceae;g__Pseudomonas	0.018202
k__Bacteria;p__Proteobacteria;c__Gammaproteobacteria;o__Pasteurellales;f__Pasteurellaceae;g__Actinobacillus	0.013722
k__Bacteria;p__Actinobacteria;c__Actinobacteria;o__Actinomycetales;f__Propionibacteriaceae;g__Propionibacterium	0.013162
k__Bacteria;p__Firmicutes;c__Clostridia;o__Clostridiales;f__Veillonellaceae;g__Veillonella	0.011201
k__Bacteria;p__Bacteroidetes;c__Bacteroidia;o__Bacteroidales;f__Porphyromonadaceae;g__Porphyromonas	0.007001
k__Bacteria;p__Firmicutes;c__Bacilli;o__Bacillales;f__Staphylococcaceae;g__Staphylococcus	0.004201
Unassigned;Other;Other;Other;Other;Other	0.00084
k__Bacteria;p__Proteobacteria;c__Betaproteobacteria;o__Burkholderiales;f__Comamonadaceae;Other	0.00056
k__Bacteria;p__Actinobacteria;c__Actinobacteria;o__Actinomycetales;f__Brevibacteriaceae;g__Brevibacterium	0.00028
**KIT PM**	
k__Bacteria;p__Proteobacteria;c__Alphaproteobacteria;o__Sphingomonadales;f__Sphingomonadaceae;g__Novosphingobium	0.695451
k__Bacteria;p__Proteobacteria;c__Alphaproteobacteria;o__Sphingomonadales;f__Sphingomonadaceae;g__Sphingomonas	0.120757
k__Bacteria;p__Proteobacteria;c__Betaproteobacteria;o__Burkholderiales;f__Comamonadaceae;g__	0.07227
k__Bacteria;p__Proteobacteria;c__Alphaproteobacteria;o__Rhizobiales;f__Bradyrhizobiaceae;g__	0.035327
k__Bacteria;p__Proteobacteria;c__Alphaproteobacteria;o__Sphingomonadales;f__Erythrobacteraceae;g__	0.0284
k__Bacteria;p__Proteobacteria;c__Alphaproteobacteria;o__Rhizobiales;f__Methylobacteriaceae;g__Methylobacterium	0.019626
k__Bacteria;p__Proteobacteria;c__Betaproteobacteria;o__Burkholderiales;f__Comamonadaceae;g__Comamonas	0.014085
k__Bacteria;p__Proteobacteria;c__Alphaproteobacteria;o__Sphingomonadales;f__Sphingomonadaceae;Other	0.008081
k__Bacteria;p__Firmicutes;c__Bacilli;o__Bacillales;f__Staphylococcaceae;g__Staphylococcus	0.001385
k__Bacteria;p__Firmicutes;c__Bacilli;o__Lactobacillales;f__Enterococcaceae;g__Enterococcus	0.000924
k__Bacteria;p__Proteobacteria;c__Alphaproteobacteria;Other;Other;Other	0.000462
k__Bacteria;p__Proteobacteria;c__Alphaproteobacteria;o__Sphingomonadales;f__Erythrobacteraceae;Other	0.000462
k__Bacteria;p__Proteobacteria;c__Gammaproteobacteria;o__Enterobacteriales;f__Enterobacteriaceae;g__	0.000462
Unassigned;Other;Other;Other;Other;Other	0.000231
k__Bacteria;p__Actinobacteria;c__Actinobacteria;o__Actinomycetales;f__Dermabacteraceae;g__Brachybacterium	0.000231
k__Bacteria;p__Cyanobacteria;c__4C0d-2;o__MLE1-12;f__;g__	0.000231
k__Bacteria;p__Firmicutes;c__Bacilli;o__Lactobacillales;f__;g__	0.000231
k__Bacteria;p__Firmicutes;c__Bacilli;o__Lactobacillales;f__Lactobacillaceae;g__Lactobacillus	0.000231
k__Bacteria;p__Proteobacteria;c__Alphaproteobacteria;o__Rhizobiales;f__Bradyrhizobiaceae;g__Bradyrhizobium	0.000231
k__Bacteria;p__Proteobacteria;c__Alphaproteobacteria;o__Rhodospirillales;f__Acetobacteraceae;g__Acetobacter	0.000231
k__Bacteria;p__Proteobacteria;c__Betaproteobacteria;o__Burkholderiales;f__Comamonadaceae;Other	0.000231
k__Bacteria;p__Proteobacteria;c__Betaproteobacteria;o__Burkholderiales;f__Comamonadaceae;g__Delftia	0.000231
k__Bacteria;p__Proteobacteria;c__Betaproteobacteria;o__Rhodocyclales;f__Rhodocyclaceae;g__	0.000231
**PCR CONTROL**	
k__Bacteria;p__Proteobacteria;c__Betaproteobacteria;o__Burkholderiales;f__Comamonadaceae;g__	0.555856
k__Bacteria;p__Proteobacteria;c__Alphaproteobacteria;o__Rhizobiales;f__Methylobacteriaceae;g__Methylobacterium	0.154918
k__Bacteria;p__Proteobacteria;c__Alphaproteobacteria;o__Sphingomonadales;f__Sphingomonadaceae;g__Sphingomonas	0.139168
k__Bacteria;p__Proteobacteria;c__Alphaproteobacteria;o__Rhizobiales;f__Methylobacteriaceae;g__	0.080717
k__Bacteria;p__Proteobacteria;c__Betaproteobacteria;o__Burkholderiales;f__Comamonadaceae;g__Comamonas	0.051636
k__Bacteria;p__Proteobacteria;c__Betaproteobacteria;o__Burkholderiales;f__Oxalobacteraceae;g__Ralstonia	0.011103
k__Bacteria;p__Proteobacteria;c__Betaproteobacteria;o__Burkholderiales;f__Comamonadaceae;Other	0.002081
k__Bacteria;p__Proteobacteria;c__Gammaproteobacteria;o__Pseudomonadales;f__Pseudomonadaceae;g__Pseudomonas	0.00103
k__Bacteria;p__Firmicutes;c__Bacilli;o__Bacillales;f__Staphylococcaceae;g__Staphylococcus	0.000842
Unassigned;Other;Other;Other;Other;Other	0.000613
k__Bacteria;p__Proteobacteria;c__Betaproteobacteria;o__Rhodocyclales;f__Rhodocyclaceae;g__	0.000613
k__Bacteria;p__Proteobacteria;c__Gammaproteobacteria;o__Enterobacteriales;f__Enterobacteriaceae;g__	0.000364
k__Bacteria;p__Firmicutes;c__Bacilli;o__Bacillales;f__Bacillaceae;g__Bacillus	0.000125
k__Bacteria;p__Proteobacteria;c__Alphaproteobacteria;o__Rhodospirillales;f__Acetobacteraceae;g__Acetobacter	0.000125
k__Bacteria;p__Proteobacteria;c__Alphaproteobacteria;o__Sphingomonadales;f__Sphingomonadaceae;g__Novosphingobium	0.000125
k__Bacteria;p__Actinobacteria;c__Actinobacteria;o__Actinomycetales;f__Dermabacteraceae;g__Brachybacterium	0.000114
k__Bacteria;p__Actinobacteria;c__Actinobacteria;o__Actinomycetales;f__Micrococcaceae;g__Micrococcus	0.000114
k__Bacteria;p__Bacteroidetes;c__Flavobacteriia;o__Flavobacteriales;f__Flavobacteriaceae;g__Capnocytophaga	0.000114
k__Bacteria;p__Firmicutes;c__Bacilli;o__Lactobacillales;f__Enterococcaceae;g__Enterococcus	0.000114
k__Bacteria;p__Proteobacteria;c__Betaproteobacteria;o__Burkholderiales;f__Comamonadaceae;g__Roseateles	0.000114
k__Bacteria;p__Proteobacteria;c__Gammaproteobacteria;o__Xanthomonadales;f__Xanthomonadaceae;g__Stenotrophomonas	0.000114

**Table 3:** Summary of OTUs detected in meconium samples only, not in negative extraction controls or negative PCR controls.

**Phyla**	**Class**	**Order**	**Family**	**Genus**	**Recovered by kit**
Actinobacteria	Actinobacteria	Actinomycetales	Unknown	Unknown	QM, PS
	Actinobacteria	Actinomycetales	Microbacteriaceae	Unknown	QM
	Actinobacteria	Actinomycetales	Microbacteriaceae	Microbacterium	QS, QM
	Actinobacteria	Actinomycetales	Micrococcaceae	Unknown	QS, PM
	Actinobacteria	Actinomycetales	Micrococcaceae	Arthrobacter	QM
	Actinobacteria	Actinomycetales	Micrococcaceae	Kocuria	QM, PM
	Actinobacteria	Actinomycetales	Nocardiaceae	Rhodococcus	QS, QM, PS, PM
	Actinobacteria	Actinomycetales	Nocardioidaceae	Unknown	QS
	Actinobacteria	Actinomycetales	Pseudonocardiaceae	Unknown	QM
	Actinobacteria	Actinomycetales	Pseudonocardiaceae	Pseudonocardia	QM
	Actinobacteria	Actinomycetales	Streptomycetaceae	Streptomyces	QM
Bacteroidetes	Flavobacteriia	Flavobacteriales	Weeksellaceae	Cloacibacterium	PM
	Saprospirae	Saprospirales	Chitinophagaceae	Sediminibacterium	PS
Cyanobacteria	Chloroplast	Streptophyta	Unknown	Unknown	QS, QM
Firmicutes	Bacilli	Bacillales	Bacillaceae	Geobacillus	QM
	Bacilli	Bacillales	Paenibacillaceae	Paenibacillus	QS, QM
	Bacilli	Bacillales	Planococcaceae	Unknown	QM, PS
	Bacilli	Gemellales	Gemellaceae	Unknown	QM
	Bacilli	Lactobacillales	Unknown	Unknown	QS, QM, PM
	Bacilli	Lactobacillales	Aerococcaceae	Marinilactibacillus	QM
	Bacilli	Lactobacillales	Lactobacillaceae	Unknown	QM
	Bacilli	Lactobacillales	Streptococcaceae	Lactococcus	QM
	Clostridia	Clostridiales	Clostridiaceae	Clostridium	QM
	Clostridia	Clostridiales	Ruminococcaceae	Unknown	QS
	Erysipelotrichi	Erysipelotrichales	Erysipelotrichaceae	Catenibacterium	QM
Gemmatimonadetes	Gemmatimonadetes	N1423WL	Unknown	Unknown	QS
Nitrospirae	Nitrospira	Nitrospirales	Nitrospiraceae	Nitrospira	QS
Planctomycetes	Planctomycetia	Planctomycetales	Planctomycetaceae	Planctomyces	QM
Proteobacteria	Alphaproteobacteria	Unknown	Unknown	Unknown	QS, PM
	Alphaproteobacteria	Caulobacterales	Caulobacteraceae	Brevundimonas	QM
	Alphaproteobacteria	Rhodobacterales	Rhodobacteraceae	Paracoccus	QS
	Alphaproteobacteria	Rhodobacterales	Rhodobacteraceae	Rhodobacter	QM
	Alphaproteobacteria	Rhodospirillales	Acetobacteraceae	Unknown	QS, QM
	Alphaproteobacteria	Rickettsiales	Unknown	Unknown	QM
	Alphaproteobacteria	Sphingomonadales	Sphingomonadaceae	Sphingobium	PS
	Betaproteobacteria	Unknown	Unknown	Unknown	QM, PS, PM
	Betaproteobacteria	Burkholderiales	Unknown	Unknown	PS
	Betaproteobacteria	Burkholderiales	Alcaligenaceae	Achromobacter	QS, QM
	Betaproteobacteria	Neisseriales	Neisseriaceae	Unknown	PS
	Betaproteobacteria	Rhodocyclales	Rhodocyclaceae	Hydrogenophilus	QM
	Betaproteobacteria	Rhodocyclales	Rhodocyclaceae	Zoogloea	PS
	Deltaproteobacteria	Bdellovibrionales	Bacteriovoracaceae	Unknown	PS
	Gammaproteobacteria	Unknown	Unknown	Unknown	QS
	Gammaproteobacteria	Alteromonadales	Chromatiaceae	Rheinheimera	PS
	Gammaproteobacteria	Enterobacteriales	Enterobacteriaceae	Unknown	QS, PS, PM
	Gammaproteobacteria	Enterobacteriales	Enterobacteriaceae	Citrobacter	PS
	Gammaproteobacteria	Enterobacteriales	Enterobacteriaceae	Erwinia	PS
	Gammaproteobacteria	Legionellales	Legionellaceae	Unknown	PS
	Gammaproteobacteria	Legionellales	Legionellaceae	Legionella	PS
	Gammaproteobacteria	Pseudomonadales	Pseudomonadaceae	Unknown	PS
	Gammaproteobacteria	Xanthomonadales	Xanthomonadaceae	Unknown	QS, QM, PS

**Figure 6:**
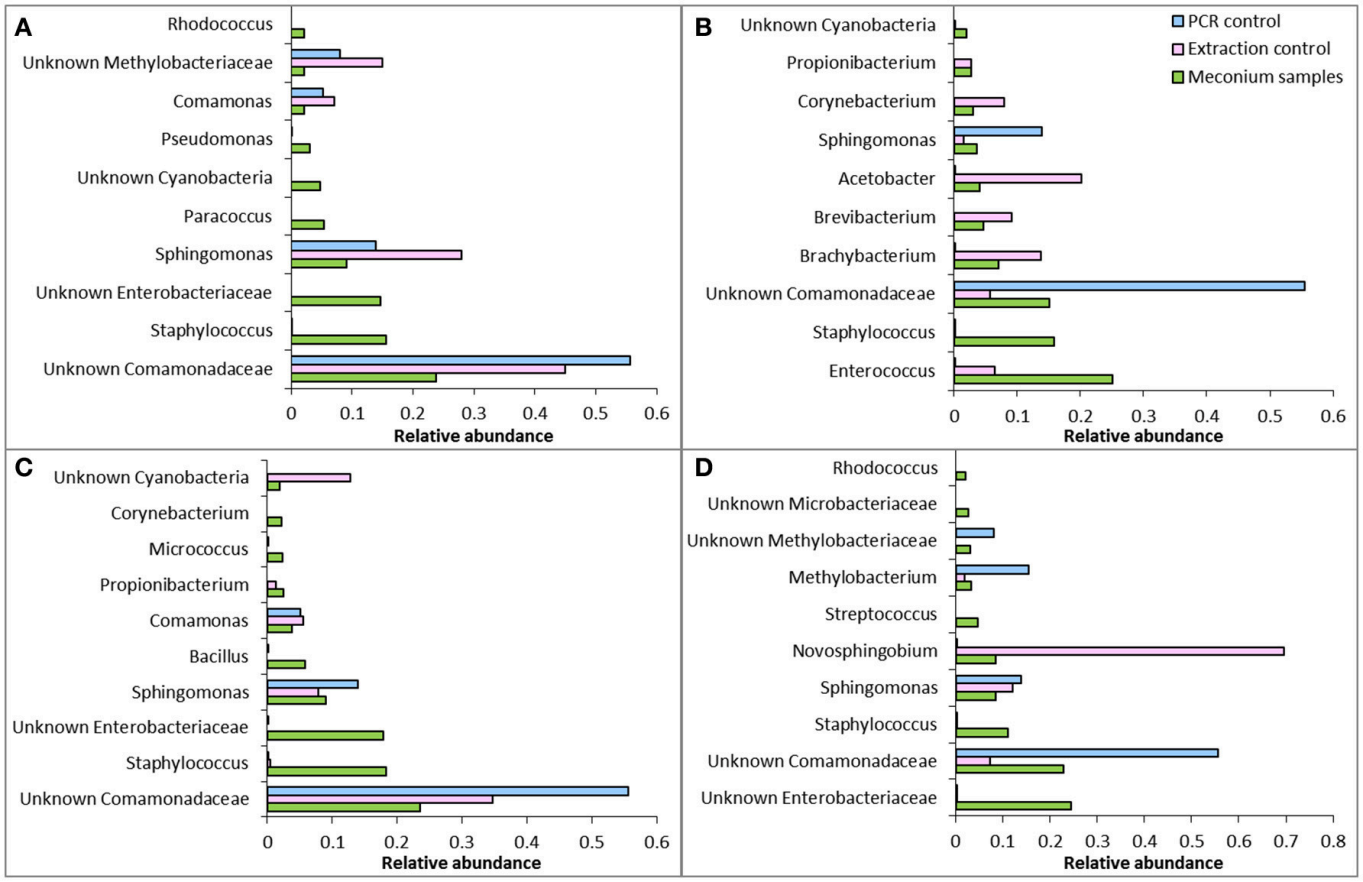
Comparison of relative abundance of the 10 most abundant OTUs in meconium samples with extraction controls and PCR controls from **(A)** kit QS, **(B)** kit QM, **(C)** kit PS, and **(D)** kit PM. Note that abundances are relative and not absolute, so a direct comparison of abundance cannot be made between meconium samples, PCR controls and extraction controls.

This study provides strong evidence that choice of DNA extraction kit impacts upon 16S rRNA gene microbial profiles generated from first-pass meconium samples. This data is in line with previous studies that have shown a high level of variation in microbiome community structure following DNA extraction with different kits (Yuan et al., 2012; Wesolowska-Andersen et al., 2014; Brooks et al., 2015; Walker et al., 2015; Vebo et al., 2016). This reinforces the need to develop a standardized, validated meconium extraction protocol so that results may be compared between studies. We have also shown that reagent and kit contamination can confound microbiome studies on meconium samples (Figure 6). Sequences generated from negative extraction and PCR controls must be taken into account when analyzing meconium microbiome profiles and data must be interpreted cautiously.

Given our data, we recommend the use of kits QM and PM for microbiome analysis of meconium. However, we caution that given the variation in OTUs recovered between kit QM and the other kits tested, results gained from use of kit QM cannot be compared to those produced with other kits in other studies.

## Summary

We compared four commonly used DNA extraction methods to assess their ability to extract DNA, overcome PCR inhibitors and analyze bacterial DNA from meconium. Our results indicate that kit PM is best able to extract microbial DNA from meconium; however, eluates require dilution to remove PCR inhibitors. We have also demonstrated a high level of variation in microbiome community structure after extraction with different kits, and the importance of controlling for external DNA contamination. Eluates generated with kit QM differed significantly from those generated with the other kits in terms of the dominant phyla and genera. The other 3 kits were consistent in terms of dominant taxa, but differed significantly in terms of low abundance OTUs.

Our results indicate that there are very low levels of human DNA in meconium relative to levels of microbial DNA and highlight the need to establish a meconium-specific sampling/extraction protocol for microbiome studies on the fetal gut. Again, we emphasize the importance of negative extraction controls for work in low biomass samples such as meconium.

## Author contributions

LS performed the experiments and data analysis and wrote the manuscript. MP and JK designed the experiments and critically edited the manuscript.

### Conflict of interest statement

The authors declare that the research was conducted in the absence of any commercial or financial relationships that could be construed as a potential conflict of interest.
